# Impact of accelerated, graduate-entry medicine courses: a comparison of profile, success, and specialty destination between graduate entrants to accelerated or standard medicine courses in UK

**DOI:** 10.1186/s12909-018-1355-3

**Published:** 2018-11-06

**Authors:** Paul Garrud, I. C. McManus

**Affiliations:** 1 Division of Medical Sciences and Graduate Entry Medicine, School of Medicine, University of Nottingham, Royal Derby Hospital, Uttoxeter Road, Derby, DE22 3DT UK; 2 Research Department for Medical Education, University College London, Gower Street, London, WC1E 6BT UK

**Keywords:** Undergraduate medical courses, Graduate entrants, 4 year vs 5 year courses, UKMED, UKCAT, GAMSAT, Demographics, Completion, Outcome measures, ARCP, Speciality choice, Validity

## Abstract

**Background:**

Little research has compared the profile, success, or specialty destinations of graduates entering UK medical schools via accelerated, 4-yr, standard 5-yr and 6-yr programmes. Four research questions directed this investigation:-

- What are the success rates for graduates entering graduate-entry vs. undergraduate medicine courses?
- How does the sociodemographic and educational profile differ between these two groups?
- Is success – in medical school and foundation training – dependent on prior degree, demographic factors, or aptitude test performance at selection?
- What specialty do graduate entry medicine students subsequently enter?

**Methods:**

The data from two cohorts of graduates entering medical school in 2007 and 2008 (*n* = 2761) in the UKMED (UK Medical Education Database) database were studied: 1445 taking 4-yr and 1150 taking 5-yr medicine courses, with smaller numbers following other programmes.

**Results:**

Completion rates for degree programmes were high at 95%, with no significant difference between programme types. 4-yr entrants were older, less likely to be from Asian communities, had lower HESA (Higher Education Statistics Agency) tariff scores, but higher UKCAT (UK Clinical Aptitude Test) and GAMSAT (Graduate Medical School Admissions Test) scores, than 5-yr entrants.

Higher GAMSAT scores, black or minority ethnicity (BME), and younger age were independent predictors of successful completion of medical school. Foundation Programme (FPAS) selection measures (EPM – educational performance measure; SJT – situational judgment test) were positively associated with female sex, but negatively with black or minority ethnicity. Higher aptitude test scores were associated with EPM and SJT, GAMSAT with EPM, UKCAT with SJT. Prior degree subject, class of degree, HESA tariff, and type of medicine programme were not related to success.

**Conclusions:**

The type of medicine programme has little effect on graduate entrant completion, or EPM or SJT scores, despite differences in student profile.

Aptitude test score has some predictive validity, as do sex, age and BME, but not prior degree subject or class. Further research is needed to disentangle the influences of BME.

UK medical schools have always accepted a proportion of entrants who had previously studied a degree in a non-medical subject, typically, but not always, a science subject. Prior to 2000 these graduate entrants studied alongside school leavers in the existing UK 5 year medicine courses.^1^ Since then, around 800 graduates annually have entered the 15 UK graduate-entry 4-year accelerated programmes, while a smaller number have continued to join the existing (Standard) 5 year programmes. The profile of graduate-entrants has also been markedly different from undergraduate programmes in terms of age and subject background, with nine of the graduate entry courses admitting students with degrees in non-science subjects. Selection criteria are also varied with different aptitude tests (e.g. United Kingdom Clinical Aptitude Test (UKCAT) or Biomedical Admissions Test (BMAT) mostly used for standard 5 year courses and GAMSAT used for 4 year Graduate courses), qualification requirements (e.g. GCSE and A-level grades for standard course entrants, but degree class mostly used for graduates applying to either four or 5 year courses, with UK degrees being classified as first class (I), upper second (2i), lower second (2ii), or third class (3) degree). Interviews are often held also, but results for the different types (MMI – multiple mini-interview, panel, assessment centres) are not available in UKMED (United Kingdom Medical Education Database).

The clear differences between four and 5 year medical courses are of interest to medical educationalists, not least because it raises the obvious question of whether abbreviated courses may generally be more cost-effective, both to students and to medical schools. Comparing the outcomes of graduates on 4 and 5 year courses is therefore of great interest, although there are reasons to believe that entrants to the two types of course may differ in a range of other factors, which may complicate the interpretation of differences. This study therefore set out to examine such questions.

Older research reported [1, 2] that graduates had a number of advantages in terms of attainment and progress at medical school compared to younger entrants with only secondary educational qualifications. However, it was unclear what exactly might be responsible for these advantages. More recent research [3] analysed the year 1 performance of entrants to twelve UK medical schools in terms of potential predictors of their attainment. A-level (Advanced level) and other school attainment measures were only available in those aged under 21, but the study did confirm the strong relationship between A-level performance and year 1 medical school assessments. There was also weaker, but incremental predictive validity for a number of other pre-entry variables: these included GCSE (General Certificate of Secondary Education) performance, scores on UKCAT, and age > 21 (who were most likely graduates). Demographic variables were also influential: in particular, men and students from non-white UK ethnic minority communities performed more poorly. Of particular interest was that UKCAT scores had a stronger relation to first year outcome in those aged > 21 than in younger entrants.

Several studies of attainment at individual UK medical schools have shown that graduate-entry students have performed comparably [4, 5] or better [6–9] than undergraduate students in common assessments during the shared full-time clinical phase of those programmes. Some studies [10–15] have attempted to identify predictors of attainment in graduate-entry programmes, with mixed conclusions, but commonly that prior academic record (e.g. secondary or tertiary educational qualifications) is a reliable predictor.

At present, therefore, there is no evidence about the relative success of graduate entrants who have gone through specific 4 year graduate-entry courses vs. conventional 5 year undergraduate medicine courses. There is also very little evidence at a national, pan-individual school level, about markers of success in these different types of course for those students with a prior degree. Two key questions concern the subject of that prior degree and its class or grade. Earlier, secondary educational record may also be an important factor in success. Age, sex, socioeconomic status, and ethnicity may also be relevant factors; and, lastly, the predictive validity of aptitude tests in graduate entrants is uncertain.

## Research questions

The principal research questions were:

1. What are the success rates for graduates entering graduate-entry vs. undergraduate medicine courses?
2. How does the sociodemographic and educational profile differ between these two groups?
3. Is success – in medical school and foundation training – dependent on prior degree, demographic factors, or aptitude test performance at selection?
4. What specialty do graduate medicine students subsequently enter?

These questions were answered using data extracted from the UK Medical Education Database (UKMED) [16].

### A note on UK medical student selection and training

Medical student selection and training in the UK is complex, and a brief summary of it is probably useful. Applicants to medical school on five-year Standard Entry courses typically apply at age 17 while at secondary school, and enter medical school at the age of 18, although entrants can be older than that, including those who are graduates. Entrants to graduate-entry courses, which usually last 4 years, already have a first degree, and therefore are aged 21 or over. Selection methods differs between UK medical schools, but many Standard Entry courses consider a candidate’s school attainment (GCSEs at age 16, AS levels at age 17 and A levels at age 18), with A levels often being taken after applications have been made, but with offers contingent on grades obtained. Graduate applicants may be selected on the basis of previous A levels, or on the basis of their degree class obtained (and in many cases, particularly in the UKMED data sets, A level results are not known for graduate applicants). When AS and A level results are known they are summarised as a Tariff Score by HESA (Higher Education Statistics Agency) which has provided data for UKMED. HESA also provides information on whether a student has taken a prior course (not medicine) at a different higher education institution (HEI). Applicants to both Standard Entry and Graduate Entry courses in many, but not all, schools will be required to take an aptitude test, either UKCAT for Standard Entry courses or GAMSAT for Graduate Entry courses. Some applicants, for various reasons, will have taken both UKCAT and GAMSAT. The present study knows only about entrants to medical schools, and no information is available about the wider population of applicants, be they those who are rejected, or have entered schools other than those in the UKMED database. Detailed information on progression of medical school entrants through their courses is not available in the present study. Applicants at the end of undergraduate training apply for Foundation Training, which lasts 2 years, known as F1 and F2. Selection into foundation posts is based on two measures, the EPM (Educational performance measure) and the SJT (Situational Judgement Test). EPM summarises performance of students *within their own medical school* (i.e. it is locally standardised), and provides either their quartile relative to other students (for the first year of usage of the EPM) or the decile (for subsequent usage). Comparison of EPMs across medical schools is not straightforward therefore, particularly as some schools provide a single EPM for all graduates, irrespective of whether they have been on a Standard Entry course, a Graduate Entry course, or other types of course (such as Gateway courses). The SJT, in contrast to the EPM, is nationally standardised, with all graduates in a year taking the same test, so that marks across different schools and courses are comparable. Performance during the F1 and F2 years (and beyond) is provided by the ARCP (Annual Review of Competence Progression) assessment, which is administered by postgraduate Deaneries where doctors are working. At the end of the F2 year, doctors apply for specialty training via schemes administered via Health Education England (HEE). Further information on the careers of the cohorts of doctors in the current study were not available beyond application to specialty training.

## Methods

The dataset was provided from the UKMED database (UKMEDP02 extract generated on 21/6/2016). See the Acknowledgment sections for details provided by the suppliers on the interpretation of these data. In accordance with statistical disclosure controls, frequencies reported are rounded as follows: 0, 1 & 2 are rounded to 0; all other frequencies are rounded to the nearest multiple of 5; percentages are suppressed where based on fewer than 22.5 individuals; averages based on fewer than 7 individuals are suppressed.

As UKMED was in Phase 1 [16], complete data were not available for the 2007 and 2008 entrants as not all entrants had completed their training (i.e. the data were right truncated in time), and not all measures were available for these particular cohorts (so that, for instance, 2007 entrants to 4-yr courses entered Foundation training before the introduction of the SJT subsequently used for selection to Foundation programmes; 2008 entrants to 6-yr courses had not yet applied for specialty training).

**Fig. 1:**
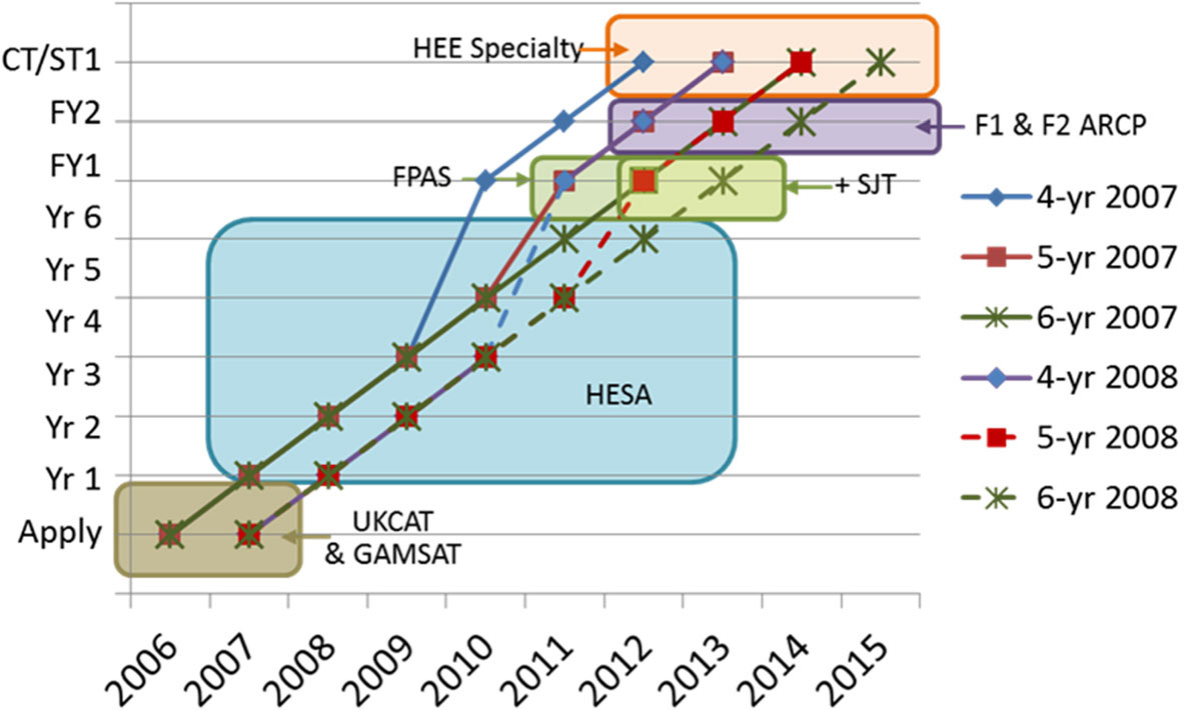
Ibry chart showing the data present in UKMED Phase 1. For details see text

Data in longitudinal cohort studies can be difficult to visualise, particularly when all data are not available for all cohorts, with truncation to both left and right due to extrinsic limitations (e.g. data were not collected or were not available before certain dates, individuals have taken different trajectories for various reasons (e.g. intercalating degrees), or individuals have not yet reached particular stages of their careers). A solution for representing such problems is what is often called the Ibry chart [17, 18], described in the nineteenth century [19], and used then, and still used, for describing the complexities of railway timetables. In the context of medical education a version of an Ibry chart was developed by one of us in 1985, although without knowing its provenance [20](p.32). Figure 1 shows a simplified Ibry chart for the present study, the horizontal axis showing calendar year, and the vertical axis the successive years of undergraduate and early postgraduate training in the UK. Different coloured lines show the trajectories of students entering in 2007 or 2008, and graduating four, five or six years later. At application, the students have information on UKCAT and/or GAMSAT (the brown box). Information on progression during medical school is provided by HESA (the blue box), and information about selection into the foundation programme (FPAS) is shown in the yellow box. ARCP results are available at the end of the F2 year and are shown in the purple box. Finally, information on choice of specialty, provided by HEE, is shown in the pale yellow box at the top. Not all students continue on these idealised paths, some delaying for a host of reasons (course failure, intercalated degrees, illness, etc) but for simplicity they are not shown in Fig. 1. Limitations of the data are shown, as an example, by the blue line for the entrants to 4-yr courses in 2007, who graduated in 2011, but FPAS results were only introduced in 2012 (the pale yellow boxes).

All records were reviewed for consistency with known graduate entry programmes, with known transfer arrangements between medical schools (e.g. Bradford-Leeds, St. Andrews-Manchester/Glasgow), and for prior degree qualification and institution. A small number in the dataset were discarded – 20 records for entrants to University of Hertfordshire that does not run a GMC (General Medical Council) primary medical qualification.

Each record was coded in terms of the type of medical programme followed.^2^ The majority comprised standard 5-year or accelerated 4-year medicine courses. In addition, small numbers of graduates appeared in the database after transfer from a previous institution, completing only 2 or 3 years of clinical medicine before graduation. In addition, some were registered for only a single year – presumably exchange students – and were excluded from analysis. Lastly, a small number of graduates enrolled on 6-year medicine programmes, either ones that provided a foundation year for those with non-science degrees, or those with a widening access remit (Gateway courses): these were combined in the analysis. Table 1 below displays the numbers in each category.

**Table 1:** Numbers of graduate entrants by type of medicine programme

Type of medicine programme	Number
4 year graduate entry	1445
Accelerated 5 yr. course	75
5 year	1150
6 year	45
Transfer to 2 yrs. clinical	15
Transfer to 3 yrs. clinical	25

Note that because of statistical disclosure controls, frequencies are rounded (see Methods)

For the purpose of analyses, only students taking a full 4-yr, full 5-yr, full 6-yr, or full but accelerated 5-yr medicine programme were included (i.e. omitting 40 who had entered medical school prior to 2007 and transferred into 2 or 3-yr clinical courses). Records were also coded by type of medical school as: Oxbridge, Russell Group (exc. Oxbridge), Non-Russell Group (exc. New medical schools), and New medical schools (e.g. Plymouth, Exeter, East Anglia).^3^

A number of summary variables were computed from the HESA data fields, viz.: age at start of course, years to complete course, success in completing course, UKCAT Total score, satisfactory F1 ARCP and F2 ARCP outcomes, GAMSAT reported degree subject,^4^ etc.

Different data were available for different years of selection to the foundation programme; 1 year had EPM (educational performance measure) in quartiles and later years in deciles: these were converted in two ways: a) to normalised deviate values (z-SJT) within each school^5^ and b) to binary values (upper half vs. lower half), and then the two measures merged separately across schools; SJT (situational judgment test) data were not available for the first 2 years completing medical school in this dataset (see Fig. 1); however, the available FPAS SJT scores (equated for the different test forms used) were first normalised within the year of testing, then merged across years.

In general, the dataset had a considerable amount of missing data. Some data were absent for known structural reasons, such as aptitude test performance since only some applicants took GAMSAT or UKCAT, but other data was missing that may have been for systematic reasons (e.g. no ARCP outcomes for students who did not complete medical school or chose not to apply for foundation training), or unsystematic ones (e.g. HESA data on prior HEI - Higher Education Institution, or HESA tariff). The approach taken, therefore, was to employ multiple imputation [21–23] with 100 replications as an SPSS procedure. As is generally recommended, the imputation used a wide range of measures, consisting of twenty variables which included demographic, admissions, and outcome variables. One should, as always, interpret the results of the imputation analyses with caution, especially where the degree of ‘missingness’ was considerable or the pattern of results differed markedly between original and pooled data. Of course, the sample size was relatively small and comprised only 2 years’ intake to medical school: student profiles and other factors may well have shifted in the last 8 years.

Statistical analyses used SPSS 24, using the t-test, oneway ANOVA, crosstabs, linear regression and logistic regression programs, as well as multiple imputation. The conventional significance level was set at 0.05, although, as always, care should be taken in interpreting results where multiple significance tests are being carried out.

## Results

Not all measures were available for all candidates, for structural and other reasons. Of the 2780 medical school entrants in the study, data were available for varying numbers of individuals. Valid Ns were: Selection measures: UKCAT (2030), GAMSAT (750) and HESA Tariff (475); Outcome measures: FPAS-EPM (1785), FPAS-SJT (710), ARCP (1825 and 2285 for F1 and F2); and Demographics: Degree class (690), NS-SEC (1645), Sex (2635) and parental degree (1425). Ns vary therefore in all analyses, with multiple imputation being used as a partial solution.

Table 2, below, shows the demographic characteristics of graduate entrants by type of medicine programme.

**Table 2:** Demographic profiles of graduate entrants by type of medicine programme

Medicine Programme type	4 year	accelerated 5 yr. course	5 year	6 year	significance
Number	1445	75	1150	45	
Mean age	24.9^b^	25.3^b^	23.9^a^	24.6^a,b^	ANOVA, F(3,2715) = 14.7, *p* < .001
Gender					
women	57.1%	54.7%	60.1%	59.6%	
men	42.9%	45.3%	39.9%	#	
Ethnicity					
white	78.8%	87.8%	70.1%	80.9%	Chi-square = 78.3, 15 df, *p* < .001
Asian	11.9%	#	20.0%	#	
black	3.3%	#	3.4%	#	
mixed	4.5%	#	2.4%	#	
Parental higher education					
yes	56.6%	50.7%	44.6%	63.8%	Chi-square = 19.2, 3 df, *p* < .001
NS-SEC					
managerial or professional	67.2%	68.0%	63.2%	54.8%	Chi-square = 49.1, 21 df, *p* < .001
intermediate occupations	13.2%	#	14.0%	#	
small employers and own account workers	1.7%	#	3.3%	#	
lower supervisory	1.4%	#	2.3%	#	
semi-routine & routine occupations	16.6%	#	17.1%	#	

# - figure suppressed due to disclosure controls

Note that because of statistical disclosure controls, frequencies are rounded and percentages sometimes omitted (see Methods). Superscripts indicate homogenous subsets (*p* < .05) for ANOVA only

As Table 2 shows, although the majority of graduates enter accelerated, 4-year medicine programmes, there is still a substantial number taking the much longer-established 5-year courses. The sex balance is broadly similar with women in a slight majority, reflecting the proportions applying [24]. However, the different types of programme vary significantly in terms of age, ethnicity, and socioeconomic status. The mean age of students taking 4-yr, accelerated, or 6-yr courses is about a year older than those taking 5-yr programmes (*p* < 0.001); of course, there is also a considerable range – from below 21 to 49 years at entry. Ethnicity also differs (*p* < 0.001), with a higher proportion of students from white communities and a lower proportion from Asian communities taking 4-yr, accelerated, and 6-yr programmes. Percentages of black students are comparable between 4-yr and 5-yr programmes. Lastly, the socioeconomic profile varies significantly (*p* < 0.001): it was similar for 4-yr and 5-yr courses, but distinctly fewer graduates entering 6 yr. courses came from managerial/professional backgrounds and more from routine/semi-routine occupations. In contrast, those from 6-yr programmes more frequently reported parents with HE (higher education) qualifications. It should be remembered that the vast majority of all the graduates will have been classified on the basis of their own occupations rather than parental – those working as (say) healthcare assistants, therefore, are likely to be classified lower than others.^6^

### Educational characteristics

Table 3, below, reports the prior educational characteristics of students entering each type of medicine programme. Since only those taking GAMSAT were asked for details of their prior degree (circa 750) the original degree characteristics data on which this table is based represent roughly 25% of the total sample.

**Table 3:** Educational profiles of graduate entrants by type of medicine programme

Medicine Programme type	4 year	accelerated 5 yr. course	5 year	6 year	significance
Highest prior qualification (HESA)					
UK first degree	83.1%	70.7%	85.2%	85.1%	Chi-square = 90.3, 24 df, *p* < .001
EU degree	1.9%	#	2.5%	#	
International degree	3.5%	#	7.9%	#	
UK higher degree	10.7%	#	3.9%	#	
Other	#	#	#	#	
GAMSAT reported degree characteristics					
Degree subject					Chi-square = 13.2, 9 df, *p* = .156
biology/life sciences	55.1%	#	63.3%	#	
other health profession	7.0%	#	#	#	
physical sciences	17.7%	#	12.9%	#	
arts, humanities, soc. sciences	20.2%	#	21.1%	#	
Degree class					
1st	18.6%	#	18.9%	#	Chi-square = 6.41, 6 df, *p* = .379
2i	59.3%	#	63.2%	#	
2ii	22.1%	#	17.9%	#	
Highest degree					
Bachelors	72.5%	#	78.4%	#	Chi-square = 18.4, 9 df, *p* = .031
Masters	15.3%	#	12.6%	#	
Doctorate	6.3%	#	#	#	
HESA Tariff					
imputed	270	284	290	276	***
original (*n* = 450)	243^a^	#^b^	312^b^	#^a^	ANOVA, F(3,467) = 8.826, *p* < .001
UKCAT					
imputed	2556	2451	2458	2474	***
original (*n* = 1920)	2,581^a^	#^b^	2,455^b^	#^a,b^	ANOVA, F(3,2009) = 45.6, *p* < .001
GAMSAT					
imputed	61.6	60.2	59.1	59.7	***
original (*n* = 740)	62.2^b^	#^a,b^	57.9^a^	#^a,b^	ANOVA, F(3,745) = 16.4, *p* < .001

# - figure suppressed due to disclosure controls, *** *p*<0.001

Note that because of statistical disclosure controls, percentages are sometimes omitted (see Methods). Superscripts indicate homogenous subsets (*p* < .05) for original data only on ANOVA tests

As can be seen in Table 3, the degree background of graduates on 4-yr and 5-yr programmes was broadly similar in terms of higher degrees, class and subject of degree, although the fuller dataset from HESA diverged from the limited information from GAMSAT takers concerning higher degrees.

The initial data about performance on aptitude tests used for selection showed that 4-yr and 6-yr entrants had lower HESA Tariffs, but higher UKCAT and GAMSAT performance compared to 5-yr entrants. Those taking accelerated 5-yr courses, in contrast, had lower UKCAT mean scores, intermediate HESA Tariffs and higher GAMSAT scores than those taking the full 5-yr courses. After imputation, all these differences were somewhat smaller, but highly significant (all *ps* < 0.001).

### Success at medical school

In the UKMED dataset at present, there are no interim performance measures for medical school. Hence two sets of data were analysed to address research question 3: successful completion of a medicine programme (variable HESA_RSN_END), and the two measures used in selection for the UK Foundation Programme (FPAS) – the Educational Performance Measure (EPM: a ranking within each medical school, based usually on performance weighted more heavily towards later years) and the Situational Judgment Test (SJT: introduced in 2012). A number of the demographic variables were simplified for these analyses: in particular, minority ethnicities were combined to one BME category (Asian, black, and mixed); although this aggregates the different proportions entering 4-yr and 5-yr courses, published evidence does not suggest major differences in UKCAT or GAMSAT scoring [14, 25].

### Completion of medicine programme

A binary variable was constructed from the HESA_RSN_END data to represent successful completion of a medicine programme or failure to complete (1 = successful completion, 0 = did not complete). Predictor variables were chosen as being likely to be of significance, or of being of pragmatic interest. Overall the successful completion rates in the different types of medicine programme were 95% (4-yr 95%, 5-yr 95%, 6-yr 92%, accelerated 5-yr 94%). However, univariate logistic regression showed that the type of medicine programme was not significantly related to successful completion (all ps > 0.6 for original and pooled data), neither was sex (*p* > 0.3), socioeconomic status (all ps > 0.18), nor HESA tariff (*p* > 0.18). In contrast, lower age starting medicine, ethnic minority status (BME), higher UKCAT Total and higher GAMSAT mean scores were all significantly related to successful completion (all ps < 0.001) in univariate regressions using the imputed data; minority ethnicity did not reach significance in the raw data. These significant factors were then entered into a multiple logistic regression using the imputed dataset. This showed that three factors reliably predicted successful completion – lower age at start of medicine course, higher GAMSAT mean score, and BME ethnicity. The results are summarised below in Table 4.

**Table 4:** Successful completion of medicine programme - multiple logistic regression (1 = successful completion, 0 = not completed)

		B	S.E.	Wald	df	Sig.	Exp(B)
Original data (*n* = 435)	GAMSAT	.069	.040	2.931	1	.087	1.072
	BME ethnicity (0 = White; 1 = BME)	.642	.477	1.810	1	.178	1.900
	Age at start in years	−.053	.048	1.201	1	.273	.948
	UKCAT	.000	.001	.034	1	.854	1.000
	Constant	−.737	2.571	.082	1	.775	.479
Pooled (*n* = 2720)	*GAMSAT*	*.080*	*.030*			*.010*	*1.083*
	*BME ethnicity* (0 = White; 1 = BME)	*.530*	*.201*			*.008*	*1.699*
	*Age at start in years*	*−.100*	*.020*			*.000*	*.905*
	UKCAT	−.000	.001			.572	1.000
	Constant	1.230	1.179			.297	3.420

Note: Exp(B) commonly known as Odds ratio is per unit change in the independent factor

Significant results (*p* < .05) are shown in italics

**Fig. 2:**
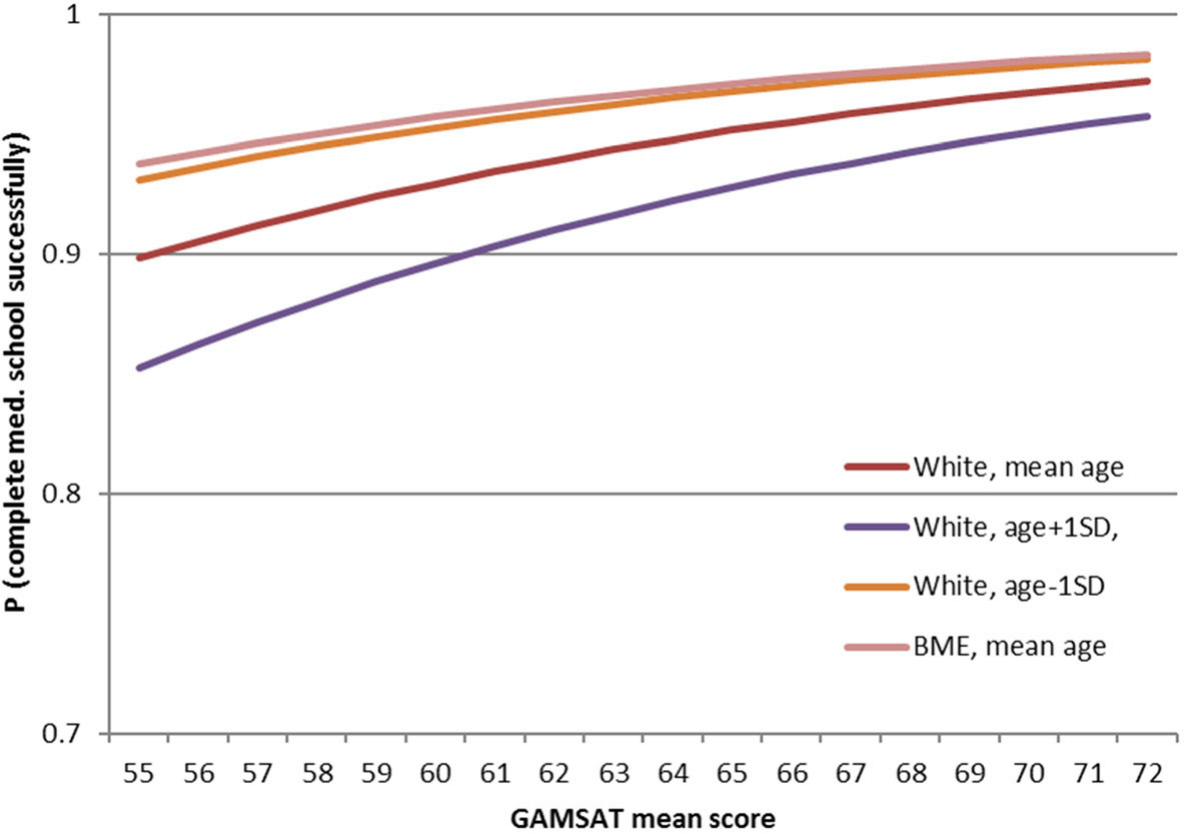
Probability of successful completion against GAMSAT mean score plotted for a student with an average UKCAT score

An alternative way of comparing the influence of GAMSAT, age at start of programme, and minority ethnicity, is to compute what improvement in the probability of successful completion would be for a one standard deviation change in the continuous variables or for a category change in the ethnicity variable. These are shown in Table 5 and Fig. 2 below.

**Table 5:** Effect of predictor factors on successful completion

Effect size for a category or 1 SD change in continuous measures		
Factor	Logits	Exp(logits)
GAMSAT	0.58	1.78
BME Ethnicity (0 = White; 1 = BME)	0.53	1.70
Age at start	−0.42	0.66

### FPAS educational performance measure (EPM)

As mentioned under methods, EPM rankings were transformed two ways - normalised within each medical school so as to combine the earlier and later rankings (quartiles, deciles respectively), or converted into a binary ranking (upper vs. lower 50%) and since EPMs were computed, in all cases, in combination with students taking 5-yr or other medicine programmes at the same school and forming part of the same graduation cohort.^7^ The normalised and binary EPM values were then merged across schools.

Significant univariate relationships^8^ were found between these normalised or binary EPM and sex, ethnicity, GAMSAT mean score, UKCAT total score, GAMSAT grouped degree subject, and GAMSAT recorded degree class (all ps = < 0.05), but not with type of medicine programme, age at start, SEC (socioeconomic classification), or medical school type (all ps > 0.1). Multiple regression using these variables as independent predictors showed that only two were significantly related to normalised EPM: Minority (BME) ethnic status was negatively related to normalised EPM, and higher GAMSAT scores were positively related to normalised EPM. Table 6 below summarises these multiple regression results. Multiple regression using binary EPM gave a slightly different pattern: BME ethnicity being negatively and GAMSAT mean score being positively related to Binary EPM in the original data (both ps < 0.001), though neither factor reached significance in the pooled data (*p* = 0.065 and 0.174 respectively).

**Table 6:** FPAS normalised EPM: Multiple regression results. Significant results (p < .05) are shown in italics

		B	S.E.	Sig.
Original data (*n* = 270)	Female sex	.196	.115	.089
	*BME ethnicity* (0 = White; 1 = BME)	*−.415*	*.137*	*.003*
	UKCAT	.000	.000	.492
	*GAMSAT*	*.050*	*.010*	*.000*
	GAMSAT Degree class	.095	.052	.068
	GAMSAT Degree subject	−.004	.068	.958
	*Constant*	*−2.825*	*.683*	*.000*
Pooled (*n* = 685)	Female sex	.210	.113	.064
	*BME ethnicity* (0 = White; 1 = BME)	*−.310*	*.139*	*.026*
	UKCAT	.000	.000	.712
	*GAMSAT*	*.024*	*.011*	*.028*
	GAMSAT Degree class	.041	.042	.327
	GAMSAT Degree subject	.016	.055	.775
	*Constant*	*−2.130*	*.707*	*.003*

**Fig. 3:**
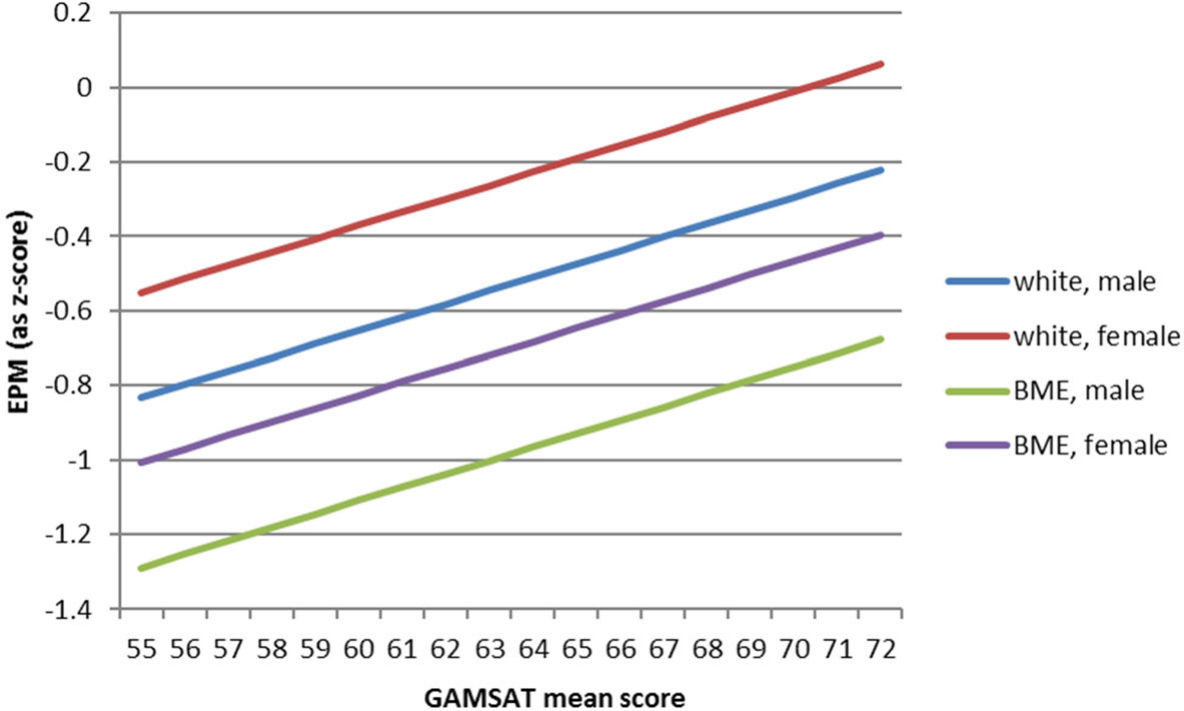
Normalised Educational Performance Measure against GAMSAT mean score for a student with 2i degree and average UKCAT score

An alternative view of this is shown in Table 7 where the effect of a category change or one SD increase in predictors can be seen, and in Fig. 3, below, the relationship of normalised EPM is plotted against GAMSAT for different ethnicities and sexes.

**Table 7:** Effect of predictor factors on normalised FPAS Educational Performance Measure

Factor	Normalised EPM (z-score)
GAMSAT (1 SD increase)	0.260
BME Ethnicity (0 = White; 1 = BME)	−0.456
Female sex	0.283

It is worth noting (and discussed later) that BME ethnicity is significantly related to lower EPM, but a higher likelihood of successful completion of medical school.

### FPAS situational judgment test (SJT)

The FPAS SJT was introduced for the first time in 2012, replacing the previous ‘white space’ questions. Comprising 70 clinical scenarios, it tests for professional attributes required for the Foundation Doctor role. Performance is reported to be normally distributed for the vast majority of applicants, with a small tail of lower scores. Analysis was of normalised SJT scores (see Method).

Again, univariate tests were carried out that identified statistically significant relationships with sex, BME ethnicity, GAMSAT, UKCAT, and GAMSAT degree class. Type of medicine programme, age at start, SEC, HESA tariff, medical school type, and GAMSAT degree subject group were not significantly related (all ps > 0.1). Multiple regression was then carried out using the significant univariate factors: this showed that female sex, white ethnicity, and higher UKCAT scores were significant predictors of the FPAS SJT score. Analysis details are shown in Table 8 below.

**Table 8:** FPAS SJT: multiple regression results. Significant results (*p* < .05) are shown in italics

		B	S.E.	Sig.
Original data (*n* = 100)	*Female sex*	*.515*	*.168*	*.003*
	*BME ethnicity* (0 = White; 1 = BME)	*−.367*	*.171*	*.034*
	UKCAT	.001	.000	.166
	*GAMSAT*	*.030*	*.015*	*.050*
	GAMSAT Degree class	−.017	.071	.808
	*Constant*	*−3.812*	*.964*	*.000*
Pooled (*n* = 750)	*Female sex*	*.262*	*.104*	*.012*
	*BME ethnicity* (0 = White; 1 = BME)	*−.314*	*.129*	*.016*
	*UKCAT*	*.001*	*.000*	*.022*
	GAMSAT	.009	.011	.403
	GAMSAT Degree class	.003	.046	.954
	*Constant*	*−2.589*	*.685*	*.000*

**Fig. 4:**
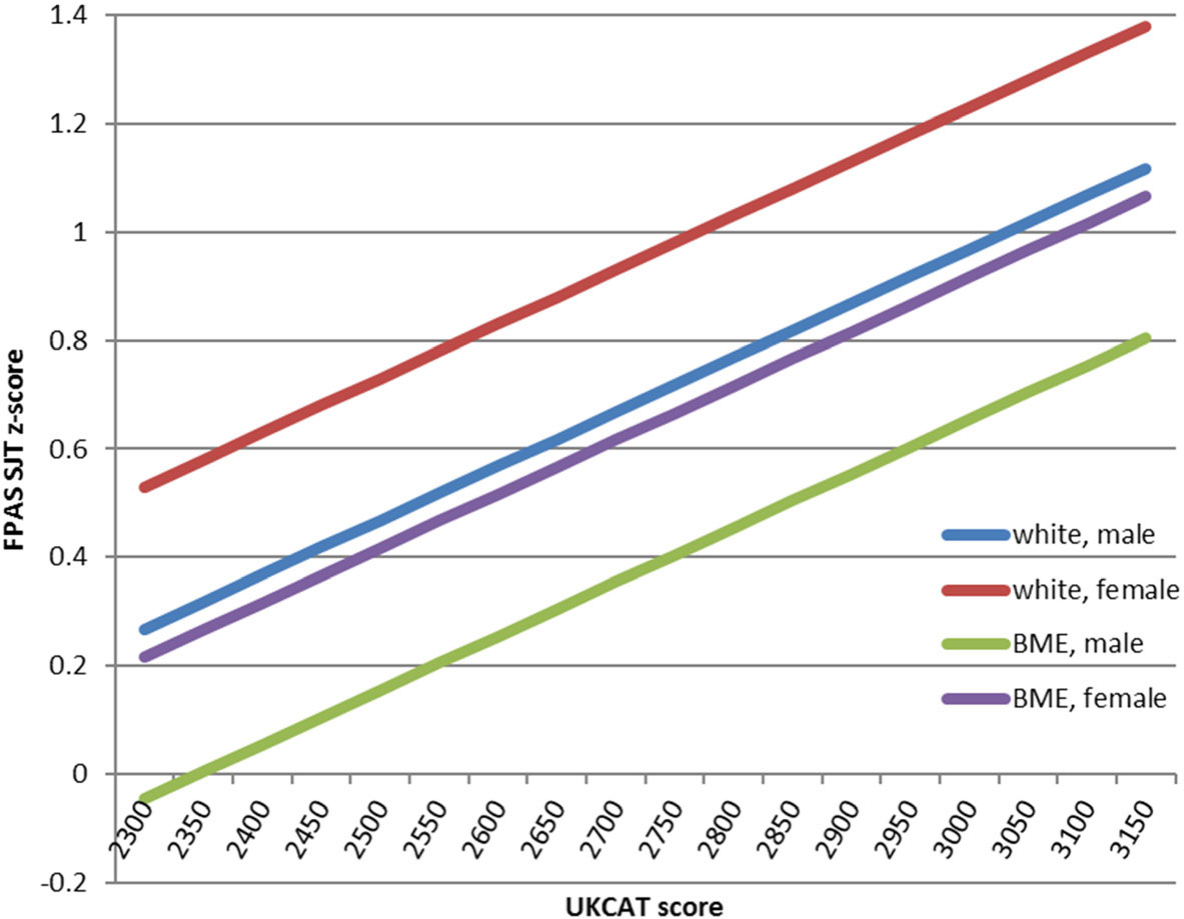
FPAS Situational Judgment Test normalised (z) score against UKCAT total score for a student with 2i degree and average GAMSAT score

An alternative view of this is shown in Table 9 where the effect of a category change or one SD increase in predictors can be seen, and in Fig. 4, below, the relationship of normalised z-SJT is plotted against UKCAT for different ethnicities and sexes.

**Table 9:** Effect of predictor factors on FPAS Situational judgment test

Factor	z-SJT score
UKCAT (1 SD increase)	0.25
BME Ethnicity (0 = White; 1 = BME)	−0.31
Female sex	0.26

### Comparison of UKCAT and GAMSAT aptitude tests

As already mentioned, both UKCAT and GAMSAT showed significant simple correlations with various outcome measures. Here we explore the details of the correlations of UKCAT and GAMSAT with the two continuous outcome measures of FPAS-EPM and FPAS-SJT (Table 10).

**Table 10:** Simple correlations of FPAS-EPM and FPAS-SJT with GAMSAT and UKCAT scores, for all graduates, and those on 4-yr and 5-yr courses

	All graduates		4-yr graduates		5-yr graduates	
	GAMSAT	UKCAT	GAMSAT	UKCAT	GAMSAT	UKCAT
FPAS-EPM	*r* = .308	*r* = .202	*r* = .240	*r* = .170	*r* = .360	*r* = .184
	*n* = 435	*n* = 1480	*n* = 285	*n* = 505	*n* = 150	*n* = 905
	*p* < .001	*p* < .001	*p* < .001	*p* < .001	*p* < .001	
FPAS-SJT	*r* = .245	*r* = .304	*r* = .339	*r* = .358	*r* = .235	*r* = .305
	*n* = 125	*n* = 640	*n* = 35	n = 45	n = 90	*n* = 545
	*p* = .006	*p* < .001	*p* = .05	*p* = .013	*p* = .028	*p* < .001

For all graduates the correlation of UKCAT and GAMSAT scores was .608 (*n* = 440, *p* < .001), for 4-yr graduates was .611 (*n* = 300, p < .001) and for the 5-yr graduates was .524 (*n* = 135, *p* < .001), showing that the two aptitude tests share much of their variance. However in Table 10, seen most clearly for all graduates, GAMSAT has a higher correlation with FPAS-EPM, whereas UKCAT has a higher correlation with FPAS-SJT.

### Completion of foundation Programme

Analysis of performance during the Foundation Programme was based on recorded ARCP (Annual Review of Competence and Progression) outcomes for year 1 (F1) and year 2 (F2). Again, this data was only available for part of the sample (e.g. F1 ARCP was missing for 4-yr graduates in 2011); for some Foundation doctors more than one outcome was recorded and so summary variables were constructed, viz.: first recorded F1 and F2 ARCP outcomes. Table 11 below shows the frequencies of each outcome against the type of medicine programme.

**Table 11:** F1 and F2 ARCP outcomes by Type of medicine programme. Note that because of statistical disclosure controls, frequencies are rounded (see Methods)

Year	Outcome	4 year	Accelerated 5 yr. course	5 year	6 year
F1	1	640	35	905	30
	5	80	5	100	5
	3 or 4	10	0	10	0
F2	1	1120	50	770	20
	5	145	10	135	0
	3 or 4	10	0	10	0

ARCP outcomes key

1. Satisfactory progress. Competences achieved as expected

3. Has not achieved competences required to progress, up to 12 months additional training required

4. Released from training programme with or without specified competences

5. Incomplete evidence provided

As can be seen above, there were very few unsatisfactory ARCPs overall, but a larger number of foundation doctors who provided incomplete evidence (all these obtained an outcome 1 later). Simple crosstabs (Chi^2^) analysis of this original data showed no significant association with type of medicine programme (all *ps* > 0.1).

### Choice of specialty after foundation training

At the end of F2, doctors can apply for specialty training (Core training or Specialty training year 1 – CT1/ST1). There are three rounds of application, shortlisting, etc., ending in appointment to a training programme. Total frequencies were calculated from the numbers of applications for specialites, and then, for larger specialties, the numbers of applications that resulted in appointments made for each specialty over the three rounds. Some specialties received only a small proportion of applications, and are omitted as statistical analysis is difficult.

Numbers of applications by specialty are shown in Table 12, divided between the 4-yr graduate entry and the full 5-yr medicine programmes.^9^ Specialties with at least 100 4-yr applicants are also analysed in terms of the success of applicants in attaining appointments.

**Table 12:** Number (%) of applications for specialty training by type of CT1/ST1 medicine programme

Specialty	4-yr Apply	5-yr Apply	Chi-square p	4-yr Appointed	5-yr Appointed	Chi-square p
ACCS (acute care common stem (acute medicine, anaesthesia, emergency medicine)	95 (6.1%)	50 (5.0%)	2.248 *p* = .134	–	–	
Anaesthetics	15	0	10.11 *p* = .001	–	–	
Core medicine	325 (20.7%)	205 (20.5%)	.856 *p* = .355	155/325 (47.7%)	85/205 (41.5%)	1.711 *p* = .191
Core psychiatry	80 (5.2%)	55 (5.7%)	.052 *p* = .819	–	–	
Core surgery	145 (9.1%)	100 (9.9%)	.019 *p* = .891	35/145 (24.1%)	30/100 (30.0%)	1.018 *p* = .313
General Practice	605 (38.2%)	395 (39.6%)	.369 *p* = .543	110/605 (18.5%)	50/395 (12.7%)	7.73 p = .005
Paediatrics	105 (6.7%)	60 (6.2%)	.779 *p* = .377	10/105 (9.5%)	5/60 (8.3%)	.787 *p* = .375
O & G (obstetrics and gynaecology)	50 (3.1%)	35 (3.6%)	.189 *p* = .664	–	–	
Radiology	50 (3.1%)	35 (3.4%)	.027 *p* = .869	–	–	
Broad-based training	30 (1.9%)	20	.000 *p* = .989	–	–	
Ophthalmology	30 (1.8%)	15	.675 *p* = .411	–	–	
Histopathology	25 (1.5%)	15	.386 *p* = .534	–	–	
Total	1585 (100%)	773 (100%)				

Note that because of statistical disclosure controls, frequencies are rounded and percentages sometimes omitted (see Methods). All chi-square statistics have 1 df

Graduates from 4-yr and 5-yr programmes showed no differences in application rates to different specialties, with the single exception of anaesthetics, for which Ns were small. For the four largest specialties in terms of applicants, only one, General Practice, showed any significant differences between those on 4-yr and 5-yr courses, 18.5% of 4-yr applicants being accepted, compared with 12.7% of 5-yr graduates (*p* = .005).

### Summary of evidence from results

The results from the analyses reported are summarised in terms of the evidence they provide in answer to the initial research questions:-.

#### What are the success rates for graduates entering graduate-entry vs. undergraduate medicine courses?

Overall successful completion did not vary significantly between 4-yr and 5-yr programmes – at 95% for both. Success rates did vary significantly with age starting medicine (younger starters being more likely to succeed), GAMSAT score (higher scorers more likely to succeed), and BME ethnicity (BME minority more likely to succeed).

#### How does the sociodemographic and educational profile differ between these two groups?

Graduates starting 5-yr courses were significantly younger than those starting 4-yr, 6-yr or accelerated 5-yr courses. They also differed significantly in terms of ethnicity (roughly twice the proportion of Asian entrants) and parental higher education (lower). Graduates entering 6-yr courses differed significantly from the other types in terms of NS-SEC (lower proportion of managerial and professional occupations). There were no significant differences in sex of students on the different types of medicine programme.

In terms of educational profile, entrants to different programmes varied significantly. Accelerated 5-yr programmes had a lower proportion of graduates with a UK first degree (70.7%), more with an EU degree (4%), and substantially more with higher degrees (18.7%) than those starting 4-yr, 5- or 6-yr courses. There were also significant differences in terms of attainment and aptitude tests: 4-yr programme entrants having lower HESA tariff, but higher scores on UKCAT and GAMSAT.

#### Is success – in medical school and foundation training – dependent on prior degree, demographic factors, or aptitude test performance at selection?

Success was evaluated in three ways – completion of medical school, FPAS educational performance measure, and FPAS situational judgment test. BME ethnicity significantly predicted a higher probability of successful completion, but lower EPM and SJT scores. GAMSAT was a significant predictor of successful completion and of FPAS EPM, in both cases higher GAMSAT predicting higher success or ranking. UKCAT did not have significant incremental validity in predicting successful completion or EPM, but higher UKCAT scores did predict SJT score. Two demographic factors were significantly related to attainment at medical school, viz.: age at start of course was negatively related to completion (older entrants being less likely to complete), and female sex was positively related to FPAS EPM ranking and SJT scores. Type of medicine programme and degree subject were not factors in any of these indicators.

#### What specialty do graduate medicine students subsequently enter?

Graduates from 4-yr programmes applied for most specialties in similar proportions, the only significant difference being that 4-yr graduates were more likely to apply for anaesthetics. Applications to General Practice were in similar proportions from 4-yr and 5-yr graduates, but 4-yr graduates were more likely to be appointed to GP training posts.

## Discussion

Abbreviated 4-yr medical school courses for graduate entry in the UK were introduced at the beginning of the current millennium, so that there are now sufficient graduates from such course to allow a comparison with graduates who have taken standard 5-year courses. The UK is unusual in having such courses, with comparison between two different routes being possible. The only comparable work we know in other countries is that in the US, where eight medical schools have introduced 3-year (as opposed to the traditional American 4-year graduate entry courses), but in a review in 2017 [26], only five of those schools had produced graduates, who totalled 51 altogether. No empirical data on differences between these graduates and traditional graduates have yet been produced (although the paper does describes three studies from the 1970s which experimented with accelerated courses). The paper also describes the 3-year programme at McMaster in Canada, and cites a 1989 study suggests that graduates are comparable to 4-year graduates (although the McMaster course is itself very different in style to other courses) [27].

The evidence derived in the present study suggests that there are many similarities between graduates entering different type of medicine programme (e.g. 95% completion rates, similar EPM rankings and SJT scores), but, perhaps, some key differences (ethnicity, tariff, aptitude scores, specialty choice). The differences are discussed in turn.

Those entering the established 5-yr programmes alongside a majority of school leavers had a higher proportion of Asian ethnicity (about 20%) and lower proportion of white ethnicity that is typical of the ethnic make-up of school leavers entering medical school as well. This difference is not easily explained by the notion that there may be a higher proportion of white students trying to do graduate entry medicine having failed to enter as a school leaver. However, it is possible that applicants choose medicine programmes where their ethnicity is already well represented.

Tariff data was missing for many of the graduates in this study, presumably because many graduate entry programmes do not require a full secondary education academic record.^10^ However, it seems clear that those entering 4-yr programmes had lower tariff scores than the other types of programme, and this is consistent with other evidence from UCAS data [15, 24]. In contrast, these same 4-yr programme entrants had significantly higher scores on both UKCAT and GAMSAT aptitude tests – in rough terms equivalent to 0.4 standard deviations, a substantial amount. They were, on average, a year older so it is possible that greater age and maturity contributed to this gain. Perhaps more likely, though, is that test results themselves (and tariff – e.g. A level requirements) influenced choice of programme to which to apply – gaining a high aptitude test score meaning a higher chance of being offered a place on a 4-yr course, a high A level tariff bringing a better chance of being accepted onto a standard 5-yr programme.

One unusual result was the disparity between the three measures of success at medical school for different ethnicities, where BME ethnicity was a predictor of a higher likelihood of completing medical school successfully, but also a predictor of poorer performance on the FPAS selection measures, EPM and SJT. The systematic review by Woolf et al. [28] reported consistently weaker academic performance by ethnic minority medical students and doctors in undergraduate and postgraduate assessments, and called for further research to track this problem and identify its causes in order to ensure fair and just methods for training and assessing future doctors. The present result may, of course, be aberrant, but it is possible that, in this group of graduate entrants to medicine, it identifies some difference in persistence as well as indications of poorer performance in the FPAS assessments.

The current results also suggest that two aptitude tests used for selection of graduates for medicine programmes (UKCAT, GAMSAT) may have some valuable predictive validity. In simple correlations each predicts success at medical school (see Table 10), but the tests predicted FPAS-EPM and FPAS-SJT differently, GAMSAT more strongly predicting EPM and UKCAT more strongly predicting SJT. GAMSAT and UKCAT differ in what they are measuring, GAMSAT containing measures of science attainment, whereas UKCAT is primarily an aptitude measure of cognitive ability, suggesting that science content helps with medical course content (i.e. EPM) whereas SJT is primarily a measure of cognitive ability. That argument is similar to that proposed by Mercer et al. [29] who in Australia showed that GAMSAT was a better predictor of attainment than UMAT in graduate entrants, UMAT also being a more of an aptitude than an attainment (knowledge) test. Our study also suggests, that when considering both GAMSAT and UKCAT as predictors of graduate entry performance, it is GAMSAT that is the major predictor, with UKCAT having little predictive effect once GAMSAT is taken into account. Academic record, in contrast to the aptitude test, did not have much predictive power in these analyses, perhaps a surprising result given the wealth of published research demonstrating this relationship amongst medical students overall (e.g. [3]), and in graduates e.g. [12]. The most likely explanation seems to be that secondary educational qualifications were mostly missing in this dataset, and that tertiary qualifications (i.e. prior degree), while predictive in some univariate analyses, did not provide significant incremental validity over the aptitude tests used. GPA (grade point average), used in most studies outside the UK, probably provides a better gradated measure than class of degree – the datum available in this study.

Specialty choice mostly did not differ between graduates from 4-yr and 5-yr medicine courses, the only exceptions being a tendency for 4-yr graduates to make more applications for anaesthetics. Amongst those applying to specialties, the only difference between 4-r and 5-yr graduates was that 4-yr graduates were more likely to be appointed to GP training. There is no obvious explanation for these differences, in a small proportion of specialties, and overall the picture is more of similarity than difference in specialty preferences.

We now turn to the methodological limitations of this preliminary study. Information was incomplete at many stages for different reasons, both systematic (e.g. FPAS SJT introduced in 2012; applicants taking only one of the two aptitude tests) and likely unsystematic (e.g. recording of secondary academic records). The use of multiple imputation, therefore, whilst substantially increasing the power of the study to detect and clarify associations and differences, should also register a reservation that imputed values are dependent on a series of assumptions about missing data. Having said that, a comparison of the results of raw (original) data and imputed data suggests that the methods give similar results in terms of effects, but the differences to a large extent are in statistical significance. As an example, consider the first three rows of Table 4, in which none of GAMSAT, BME ethnicity and Age are significant predictors in the raw data, but all three are significant in the imputed data. However the effects are similar in size (e.g. the effect of GAMSAT is .069 (se .040) in the raw data and .080 (se .030) in the imputed data, the effect sizes being very similar. The conclusion has to be that complete cases in the original data are insufficient to have adequate power to reach significance, but the imputed data find a similar effect, but one which is highly significant. That provides confidence that multiple imputation as a method is robust and not providing misleading results.

FPAS SJT data was absent for the 2007 entry to 4-yr courses, but not for 5-yr entrants that year: the cohort and course type may have influenced how those students might have performed had they taken the SJT. Similarly, although about half of those who took GAMSAT also took the UKCAT test, it is likely that which test an applicant chooses to take is closely related to their preferred medical schools and programmes. The different sources and types of data in the UKMED database most likely vary in reliability. Test scores and objectively recorded information such as UKCAT, FPAS, and registration on university course, can be viewed as reliable, self-reported information less so. Another limitation is the sample studied – entrants to UK medicine courses in 2007 and 2008. Though the profile of those entrants may have stabilised since the start of graduate entry 4-yr courses in 2001, other changes may have occurred in the subsequent cohorts – for example, the 2012 intake was the first to include graduates who had paid £9 K per annum tuition fees in England; selection criteria and admission test performance have changed in some cases as well. And, above all, those choosing to apply (and enter) medicine as graduates are likely to be highly self-selected.

## Conclusions

Our analyses have implications for selection, education and training in medicine in UK. The evident conclusion is that performance of graduates going through the dedicated 4-yr graduate entry medicine courses is broadly very comparable with graduates taking other types of programme, standard 5-yr programmes in particular. There is additional evidence that minority ethnicity status is an influential factor – though this study suggests that while performing, on average, more poorly in FPAS, students from BME ethnicity are more likely to complete their primary medical qualification. Age and sex are also indicated as influential demographic factors, though these characteristics (age, sex, ethnicity) are all protected under the UK Equality Act [30]. In this study one aptitude test (GAMSAT) seems to have a stronger relationship to medical school outcomes than the other common test (UKCAT), though one should note that the third test employed in the UK (BMAT) was not examined as those data are not currently available in the UKMED database. Though not part of the initial research questions, the relationship between performance on these two aptitude tests remains of interest: one is in part curriculum-based (GAMSAT), has greater test time (5.5 h), and is more expensive (£210 in 2016); the other is not curriculum-based, takes a shorter time (2 h), costs less and operates a bursary scheme, all of which may explain some of the differences.

In its first phase, use of the UKMED database has enabled us to characterise students starting medicine as graduates, examine their progress at medical school, in foundation training and their choice of specialty, in a national study embracing all UK medical schools. Such a study would have been almost entirely impossible before the establishment of UKMED, and shows the strength of the database. Further development of this resource in its second stage will allow even more elaborate questions to be answered more robustly.

## Abbreviations

ACCS: Acute Care Common Stem
A-level: Advanced level secondary education qualification
ANOVA: Analysis of Variance
ARCP: Annual Review of Competence and Progression
BMAT: Biomedical Admissions Test
BME: Black and minority ethnicity
CT1: Core training year 1
EPM: Educational Performance Measure
F1: Foundation Programme year 1
F2: Foundation Programme year 2
FPAS: Foundation Programme Application System
GAMSAT: Graduate Medical School Admissions Test
GCSE: General Certificate of Secondary Education
GMC: General Medical Council
GP: General Practitioner (or General Practice)
GPA: Grade Point Average
HE: Higher Education
HEE: Health Education England
HEI: Higher Education Institution
HESA: Higher Education Statistics Agency
MMI: Multiple Mini-Interview
NS-SEC: National Statistics Socioeconomic Classification
O & G: Obstetrics and Gynaecology
SD: Standard deviation
SEC: Socioeconomic Classification
SJT: Situational Judgment Test
SPSS: Statistical Package for the Social Sciences
ST1: Speciality Training Year 1
UKCAT: UK Clinical Aptitude Test
UKMED: UK Medical Education Database

## Footnotes

1 The terms 5 year and 6 year are potentially confusing. Traditionally, all UK undergraduate medical schools had 5 years of medical training (perhaps 2 years of basic medical science and 3 years of clinical training), but some students would take an additional year to intercalate a BSc or equivalent degree in a science or other subject. Traditional courses taking 6 years therefore have only 5 years of actual medical training. In recent years Gateway and similar courses have developed where students have an additional year of basic medical sciences, and these are genuinely 6 years of actual medical training. The graduate entry courses are also genuinely 4 years of actual medical training, usually consisting of 1 year of basic medical sciences with 3 years of clinical training.

2 This was not straightforward since some universities do not use different HESA codes for their different medicine programmes. Thus, for some student records, inspection of the progress through each year of programme (HESA PRG) was required to assign the type of programme.

3 Oxbridge = Oxford & Cambridge; Russell Group = 24 highly selective, research intensive UK universities; New medical schools = 9 UK medical schools established between 1997 and 2005.

4 Reported degree subjects were first coded using the GAMSAT coding scheme and then combined into four categories, viz.: biological and life sciences, health professional qualifications (e.g nursing), physical sciences (inc. maths, IT & engineering), and a final combination of arts, humanities and social sciences (inc. psychology).

5 This was done for two reasons: a) because EPM quartiles/deciles were assigned for graduates in all cases as part of a graduating cohort that included non-graduates – hence graduates were not equally distributed across all quartiles/deciles; b) in order to combine quartile and decile measures.

6 UCAS instructions to applicants say: “Please state the occupation of the highest-earning family member of the household in which you live. If he or she is retired or unemployed, give their most recent occupation.”

7 It’s also worth noting that EPM is calculated differently within each school as well. EPMs are typically calculated for all students in a cohort at a particular medical school. However if a school is entirely graduate entry then there should be 10% of students in each decile, with a median of 50%. That will still apply if a course has both graduate entrants and non-graduate entrants as all are ranked together, but if, say, the graduates are systematically better than the non-graduates, then their median rank will be higher than 50%. We have therefore rescaled all EPMs for the graduates in this study so that the deciles are indeed true deciles for the graduates themselves.

8 Linear regression for continuous variables, analysis of variance for categorical variables.

9 6-yr and accelerated 5-yr programmes were not included as the numbers were very small.

10 Indeed it is possible that some reported only those A levels or Highers required by different courses - e.g. chemistry.
